# *Notes from the Field:* Trends in Gabapentin Detection and Involvement in Drug Overdose Deaths — 23 States and the District of Columbia, 2019–2020

**DOI:** 10.15585/mmwr.mm7119a3

**Published:** 2022-05-13

**Authors:** Christine L. Mattson, Farnaz Chowdhury, Thomas P. Gilson

**Affiliations:** ^1^Division of Overdose Prevention, National Center for Injury Prevention and Control, CDC; ^2^Peers and Partners, Inc., Atlanta, Georgia; ^3^Cuyahoga County Medical Examiner’s Office, Cleveland, Ohio.

Gabapentin is an anticonvulsant medication, which is also approved by the Food and Drug Administration to treat postherpetic neuralgia, a painful complication of shingles, which results from reactivation of the varicella zoster virus.*^,†^ Gabapentin is commonly used off-label to treat neuropathic pain (*1*). Gabapentin prescribing has steadily increased in recent years, and in 2019, 69 million gabapentin prescriptions were dispensed in the United States, making it the seventh most commonly prescribed medication nationally.^§ ^Although gabapentin is generally considered safe and is infrequently associated with overdose on its own, when used with other central nervous system depressants such as opioids, there is risk for respiratory depression, potentially resulting in death (*2*).^¶^

Gabapentin can be used to potentiate illicit opioids; data indicate gabapentin exposures associated with intentional abuse, misuse, or unknown exposures reported to U.S. poison centers increased by 104% from 2013 to 2017 (*3*). However, less is known about the drug’s role in fatal overdoses (*4*). To assess quarterly trends in gabapentin-involved overdose deaths of unintentional or undetermined intent during 2019**–**2020, CDC analyzed data from the State Unintentional Drug Overdose Reporting System (SUDORS) in 23 states and the District of Columbia.** SUDORS requires jurisdictions to abstract data from death certificates and medical examiner or coroner reports, including postmortem toxicology results, which identify detected drugs and those determined to cause death (referred to as involved^††^).

Data on 62,652 overdose deaths that occurred during 2019**–**2020 in the 24 jurisdictions were entered in SUDORS; among 58,362 deaths with documented toxicology results, a total of 5,687 (9.7%) had gabapentin detected on postmortem toxicology. Gabapentin-involved deaths occurred in 2,975 of 5,687 decedents (52.3%) with a positive gabapentin test result. Across the study period, the demographic characteristics of decedents remained largely similar. Most gabapentin-involved overdose deaths occurred among non-Hispanic White persons (83.2%) and persons aged 35–54 years (52.2%); gabapentin-involved overdose deaths occurred with approximately equal frequency among men (49.7%) and women (50.3%).

During the second quarter of 2020, the number of deaths reported with gabapentin detected (959) approximately doubled compared with the first quarter of 2019 (449) (Figure); in the fourth quarter of 2020, 801 deaths with gabapentin detected occurred. Among deaths where gabapentin was detected, 49.4% were gabapentin-involved during the first quarter of 2019; this percentage increased to 55.1% during the fourth quarter of 2020.

**FIGURE:**
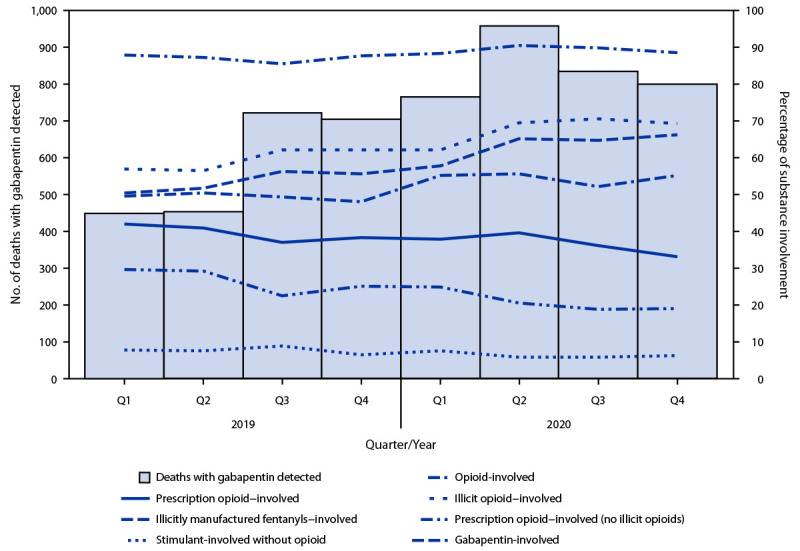
Quarterly trends in gabapentin detection and involvement of gabapentin and other substances in drug overdose deaths among decedents in whom gabapentin was detected, by substance involvement* — 23 states and the District of Columbia,^†^ 2019–2020 **Abbreviation:** Q = quarter. * Substances determined to have caused death. When nonspecific terminology was used in an overdose cause of death statement (e.g., multidrug overdose), all drugs detected in postmortem toxicology were included as involved in the death. For example, if the cause of death was “multidrug overdose,” and toxicology results were positive for five drugs, all five were classified as involved. ^†^ Twenty-three states (Alaska, Connecticut, Delaware, Georgia, Illinois, Maine, Massachusetts, Minnesota, Missouri, Nevada, New Hampshire, New Jersey, New Mexico, North Carolina, Oklahoma, Pennsylvania, Rhode Island, Tennessee, Utah, Vermont, Virginia, Washington, and West Virginia) and the District of Columbia.

The percentage of deaths with gabapentin detected that were opioid-involved remained consistently high, ranging from 85% to 90%. Illicit opioid-involved deaths accounted for 56.8% of overdose deaths with gabapentin detected in the first quarter of 2019 and 69.2% in the last quarter of 2020; this increase was largely driven by illicitly manufactured fentanyl and fentanyl analogs. The percentage of deaths that involved a prescription opioid declined from 41.9% of deaths with gabapentin detected in the first quarter of 2019 to 33.0% during the last quarter of 2020. The percentage of deaths with gabapentin detected that involved a stimulant (in the absence of opioid coinvolvement) was low and largely stable, ranging from 6% to 9%.

During 2019–2020, gabapentin detection and involvement in fatal drug overdoses increased, appearing to follow the rising trend in overall overdose deaths during the COVID-19 pandemic. Overall increases were largely driven by increases in synthetic opioids such as illicitly manufactured fentanyls and likely exacerbated by the social and economic consequences of the pandemic. Nearly 90% of drug overdose deaths in which gabapentin was detected also involved an opioid, particularly (and increasingly) illicitly manufactured fentanyls. Although gabapentin testing is recommended as part of comprehensive postmortem toxicology testing protocols for drug overdose death investigations in the United States, gabapentin is not included in the list of substances recommended in an adequate analyte panel (*5*) and is not uniformly included on death certificates by some certifiers; therefore, overdose deaths involving gabapentin or with gabapentin detected are likely underestimated. Routine gabapentin testing, as part of comprehensive postmortem toxicology testing protocols for drug overdose death investigations, could further elucidate its role in drug overdose deaths. Despite the lack of uniform testing, gabapentin detection and involvement in overdose deaths increased during 2019–2020. These findings highlight the dangers of polysubstance use, particularly co-use of gabapentin and illicit opioids. Persons who use illicit opioids with gabapentin should be educated about the increased risk for respiratory depression and death.

## Footnotes

* https://www.accessdata.fda.gov/drugsatfda_docs/label/2017/020235s064_020882s047_021129s046lbl.pdf

†
https://effectivehealthcare.ahrq.gov/products/nonopioid-chronic-pain/research

§ https://www.iqvia.com/insights/the-iqvia-institute/reports/medicine-spending-and-affordability-in-the-us https://www.fda.gov/drugs/drug-safety-and-availability/fda-warns-about-serious-breathing-problems-seizure-and-nerve-pain-medicines-gabapentin-neurontin

** Data are reported to SUDORS in half-year increments (January–June and July–December) based on when deaths occur. Twenty-three states (Alaska, Connecticut, Delaware, Georgia, Illinois, Maine, Massachusetts, Minnesota, Missouri, Nevada, New Hampshire, New Jersey, New Mexico, North Carolina, Oklahoma, Pennsylvania, Rhode Island, Tennessee, Utah, Vermont, Virginia, Washington, and West Virginia) and the District of Columbia submitted complete data at the time of analysis and were included in this report. Data for this report were downloaded on October 25, 2021, and might differ from other reports because death data might be updated over time, and SUDORS supplements death certificate data with medical examiner and coroner reports.

†† When nonspecific terminology was used in an overdose cause of death statement (e.g., “multidrug overdose”), all drugs detected in postmortem toxicology were included as involved in the death. For example, if the cause of death was “multidrug overdose” and toxicology results were positive for five drugs, all five were classified as involved.

## References

[1] Wiffen PJ, Derry S, Bell RF, Gabapentin for chronic neuropathic pain in adults. Cochrane Database Syst Rev 2017;6:CD007938. 10.1002/14651858.CD007938.pub4 28597471 PMC6452908

[2] Gomes T, Juurlink DN, Antoniou T, Mamdani MM, Paterson JM, van den Brink W. Gabapentin, opioids, and the risk of opioid-related death: a population-based nested case-control study. PLoS Med 2017;14:e1002396. 10.1371/journal.pmed.1002396 28972983 PMC5626029

[3] Reynolds K, Kaufman R, Korenoski A, Fennimore L, Shulman J, Lynch M. Trends in gabapentin and baclofen exposures reported to U.S. poison centers. Clin Toxicol (Phila) 2020;58:763–72. 10.1080/15563650.2019.1687902 31786961

[4] Finlayson G, Chavarria M, Chang S, Gabapentin in mixed drug fatalities: does this frequent analyte deserve more attention? Acad Forensic Pathol 2017;7:99–111. 10.23907/2017.012 31239962 PMC6474478

[5] Davis GG, Cadwallader AB, Fligner CL, Position paper: recommendations for the investigation, diagnosis, and certification of deaths related to opioids and other drugs. Am J Forensic Med Pathol 2020;41:152–9. 10.1097/PAF.0000000000000550 32404634

