# *Erratum:* Vol. 71, No. 19

**DOI:** 10.15585/mmwr.mm7313a4

**Published:** 2024-04-04

In the report, “Notes from the Field: Trends in Gabapentin Detection and Involvement in Drug Overdose Deaths — 23 States and the District of Columbia, 2019–2020,” on page 664, in the fifth paragraph, the first sentence should have read, “The percentage of deaths **with gabapentin detected that were opioid-involved** remained consistently high, ranging from 85% to 90%.”

